# Identification and Characterization of a Novel Prophage Lysin against *Streptococcus dysgalactiae*

**DOI:** 10.3390/molecules29143411

**Published:** 2024-07-20

**Authors:** Linan Xu, Xingshuai Li, Xiangpeng Yang, Yuzhong Zhao, Jianrui Niu, Shijin Jiang, Junfei Ma, Xinglin Zhang

**Affiliations:** 1 Department of Preventive Veterinary Medicine, College of Veterinary Medicine, Shandong Agricultural University, Tai’an 271018, China; sklp001@lyu.edu.cn (L.X.);; 2 College of Agriculture and Forestry, Linyi University, Linyi 276005, China

**Keywords:** prophage lysin, Lys1644, *Streptococcus dysgalactiae*, synergistic bacteriostasis

## Abstract

*Streptococcus dysgalactiae* infection can cause bovine mastitis and lead to huge economic losses for the dairy industry. The abuse of antibiotics has resulted in growing drug resistance of *S. dysgalactiae,* which causes hard-to-treat infections. Bacteriophage lysin, as a novel antibacterial agent, has great potential for application against drug-resistant gram-positive bacteria. However, few studies have been conducted on the prophage lysin of *S. dysgalactiae*. In this study, we mined a novel prophage lysin, named Lys1644, from a clinical *S. dysgalactiae* isolate by genome sequencing and bioinformatic analysis. Lys1644 was expressed and purified, and the lytic activity, antibacterial spectrum, optimal pH and temperature, lytic activity in milk in vitro, and synergistic bacteriostasis with antibiotics were assessed. The Lys1644 prophage lysin showed high bacteriolysis activity specifically on *S. dysgalactiae*, which resulted in CFU 100-fold reduction in milk. Moreover, Lys1644 maintained high activity over a wide pH range (pH 5–10) and a wide temperature range (4–42 °C). Synergistic bacteriostatic experiments showed that the combination of low-dose Lys1644 (50 μg/mL) with a subinhibitory concentration of aminoglycoside antibiotics (kanamycin or spectinomycin) can completely inhibit bacterial growth, suggesting that the combination of Lys1644 and antibiotics could be an effective therapeutic strategy against *S. dysgalactiae* infection.

## 1. Introduction

Bovine mastitis, mainly caused by microorganisms invading mammary tissue, is one of the most common diseases in the global dairy farming, resulting in huge economic losses for the dairy industry [1]. *Streptococcus dysgalactiae* is one of the most prevalent pathogens in dairy farming, which can cause streptococcal mastitis, endometritis in dairy cows, and skin injury, as well as meningitis in humans [2,3]. Antimicrobials are widely used to prevent and control bovine mastitis. However, the overuse of antibiotics has led to the development of bacterial resistance [4,5]. In previous studies, clinical strains of *S. dysgalactiae* have exhibited resistance in many areas to kanamycin, erythromycin, streptomycin, tetracycline, and lactamases [6,7,8]. These resistant strains have the potential to spread to humans through the food chain, thereby presenting a notable threat to human health [9]. Consequently, the exploration of novel antimicrobial agents to replace antibiotics holds importance in achieving antibiotics-free farming within the dairy industry and the protection of the development of the livestock industry and human health.

Bacteriophage lysin, a cell wall hydrolase, is expressed during the late stage of bacteriophage infection. Its specificity to host bacteria, potent antibacterial properties, low capacity to produce drug resistance, and potential for synergistic effects with other antibacterial agents position it as a promising novel antibacterial drug [10,11]. Phage lysin can be sourced from virulent phages or prophages. The prophage genome is an excellent source of *Streptococcus*, for which it is difficult to isolate virulent phages [12]. Previous studies have reported lysins against other streptococci, such as PlyC and PlyPy against *Streptococcus pyogenes* [13,14], PlyGBS and B30 against *Streptococcus agalactiae* [15,16], and Pal and Cpl-1 against *Streptococcus pneumoniae* [17,18]. The lysins PlySK1249 [19], CF-301 [20], λSA2, B30 [21], Ply700 [22], and PlySs2 [23], derived from *Streptococcus suis*, *S. agalactiae*, group B *Streptococcus,* and *Streptococcus uberis*, as well as the engineered chimeric enzymes ClyR [24] and Ply187N-V12C [25], have been reported to exhibit lytic activity against *S. dysgalactiae*. These lysins typically demonstrate a broad lytic spectrum within *Streptococcus*, and also show certain lytic effects on *S. agalactiae*, *S. uberis,* and *S. pyogenes*.

In this study, we mined a novel lysin that specifically lyses *S. dysgalactiae*, Lys1644, from a prophage genome of *S. dysgalactiae* and explored its bactericidal activity and its synergistic effect with antibiotics.

## 2. Results

### 2.1. The Gene Lys1644 Encoding Lysin Exists in the Prophage pp6 Genome of S. dysgalactiae Lu24

**Figure 1:**
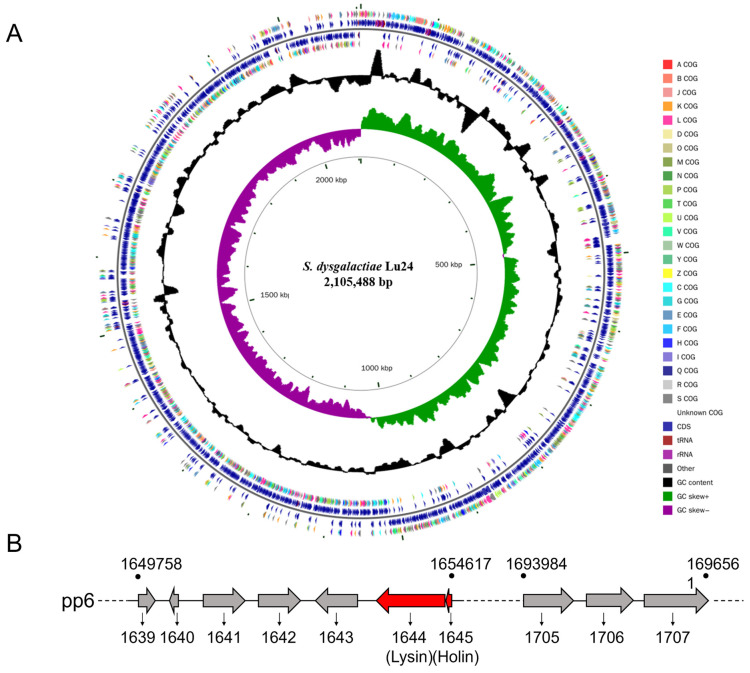
Genome sequencing circle of *S. dysgalactiae* Lu24 (**A**) and the gene cluster of the prophage pp6 (**B**). These genes, *pp6_l644* and *pp6_1645,* are predicted to encode lysin and holin, respectively.

To obtain the genome sequence of the prophage, whole-genome sequencing of *S. dysgalactiae* Lu24 (NCBI sequence ID: NZ_CP142018.1) was performed. The length of the whole genome of *S. dysgalactiae* Lu24 was 2,105,488 bp, encoding about 2117 genes (Figure 1A). As shown in Figure 1B, the prophage pp6 was about 46,800 bp in length, located between 1,649,758 bp and 1,696,561 bp of the *S. dysgalactiae* Lu24 genome. Among the 69 genes of the prophage, a binary lytic system containing lysin encoded by *pp6-1644* (named *Lys1644;* amino acid sequence is shown in Table S3) and holin encoded by *pp6-1645* was identified.

### 2.2. The Lysin Lys1644 Containing Two Lytic Domains Was Successfully Expressed and Purified

**Figure 2:**
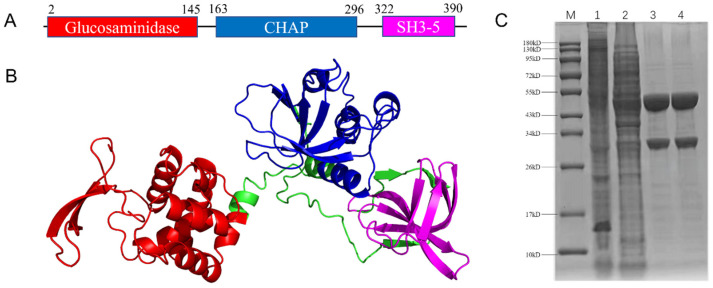
Structure analysis and expression of Lys1644. (**A**) The structural domain of Lys1644 predicted by Intropro server. (**B**) The 3D structure of Lys1644 predicted by AlphaFold2. The Glucosaminidase domain is in red, the CHAP domain is in blue, the SH3-5 domain is in purple, and the linker is in green. (**C**) Expression and purification of Lys1644 visualized by SDS-PAGE. M: protein marker; 1, total proteins of BL21 DE3 (pEC); 2, total proteins of BL21 DE3 (pEC-*Lys1644*); 3,4, purification of lys1644.

The domain prediction conducted using the Interpro sever (Figure 2A) revealed that Lys1644 contained a glucosaminidase domain (1–145 aa), a cysteine histidine-dependent amidohydrolase/peptidase (CHAP) domain (163–296 aa), and a SH3-5 domain (322–390 aa). Among them, glucosaminidase could cleave the 1,4-glycoside bond between N-acetylglucosamine and N-acetylmuramic acid, while CHAP could cleave the amide bond between glycans and peptides. The SH3-5 domain located at the C-terminus exhibited a binding function to the cell wall. The structure prediction by Alphafold2 (Figure 2B) showed that the three domains (glucosaminidase: red, CHAP: blue; SH3-5: purple) exhibited relatively independent spatial units interconnected by linkers (green). Although the linker regions displayed relatively low prediction confidence, the three domains showed very high prediction confidence (>90). Further, the SDS-PAGE analysis (Figure 2C) showed an additional protein band of 45 kD in BL21 DE3 (pEC-*Lys1644*, Lane 2) compared to BL21 DE3 (pEC, Lane1). Through affinity chromatography, the Lys1644 protein was successfully purified (Lane 3,4). After purification, there was a coarse protein band at 34 kD, which we suspected might have been caused by protein degradation of Lys1644.

### 2.3. Lys1644 Is a Specific Lysin against S. dysgalactiae

**Figure 3:**
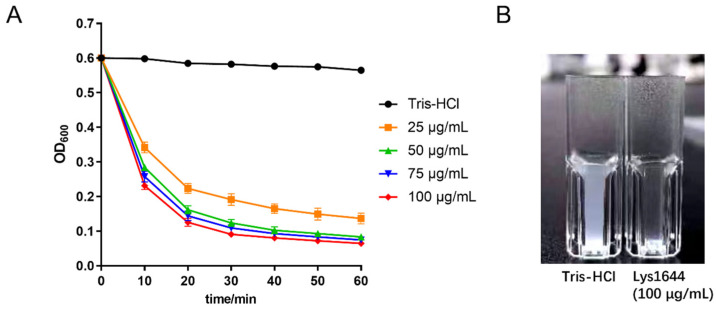
Lytic activity of Lys1644 against *S. dysgalactiae*. (**A**) Lysis curve of Lys1644 under different concentrations (25, 50, 75, 100 μg/mL). (**B**) Bacterial turbidity after lytic action of Lys1644 at 60 min. Tris-HCl buffer was used as a control.

The lytic activity of Lys1644 was assessed using *S. dysgalactiae* Lu24. As shown in Figure 3A, the Lys1644 exhibited dose-dependent lytic efficacy of Lys1644 against *S. dysgalactiae* Lu24. When the concentration of Lys1644 reached 50 μg/mL, the cleavage efficacy was already good. When the concentration exceeded 50 μg/mL, there was no discernible variation in lytic activity against *S. dysgalactiae.* The turbidity of bacteria exhibited a significant decrease following the application of Lys1644 for 1 h (Figure 3B). In addition, the activity spectrum of Lys1644 was determined. The results (Table 1) demonstrated that Lys1644 only exhibited lytic activity against *S. dysgalactiae* (3/3) while displaying no lytic activity against other *Streptococcus* species, such as *S. uberis*, *S. agalactiae*, *S. infantarius*, *S. suis*, *S. pneumoniae*, and *S. pyogenes*, nor against other bacteria, such as *L. monocytogenes*, *S. aureus*, *E. faecalis*, *E. faecium*, and *E. coli.* It is evident that Lys1644 possesses specific lysis properties targeting only *S. dysgalactiae*.

**Figure 4:**
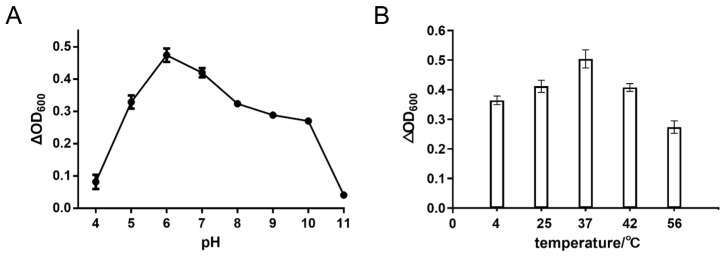
Determination of optimal pH (**A**) and temperature (**B**) of Lys1644.

Further, the optional pH and temperature of Lys1644 were tested according to the decrease in OD_600_ of *S. dysgalactiae* GS4-4 cells treated with Lys1644 at different pHs and temperatures. The enzymatic activity of Lys1644 was found to be minimal at pH 4 and 11, while it exhibited high activity across a broad pH range (pH 5–10), with an optimal pH of 6 (Figure 4A). In addition, the enzyme Lys1644 exhibited the highest activity at 37 °C, while displaying lower activity at temperatures of 4, 25, 42, and 56 °C (Figure 4B).

### 2.4. Lys1644 Has Fairly Strong Lytic Activity in Milk

**Figure 5:**
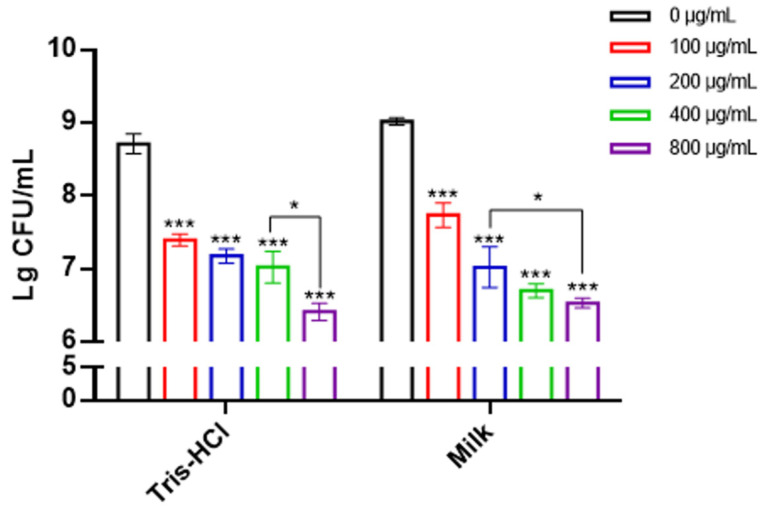
Lytic activity of Lys1644 in milk. Tris-HCl buffer was used as a control. *** *p*  ≤  0.001, * 0.05 < *p* < 0.1.

Considering the practical application of lysin, the lytic activity of Lys1644 in milk was determined. As shown in Figure 5, the concentration of Lys1644 at 100 μg/mL exhibited a reduction in *S. dysgalactiae* levels in milk by about an order of magnitude. When the concentration reached 800 μg/mL, Lys1644 exhibited a reduction in *S. dysgalactiae* in milk by about two orders of magnitude. The lytic activity of 1ys1644 in milk is comparable to that observed in Tris-HCl buffer. This result indicates that Lys1644 has practical value in the treatment of *S. dysgalactiae* infection.

### 2.5. Lys1644 Has a Synergistic Effect with Antibiotics

**Figure 6:**
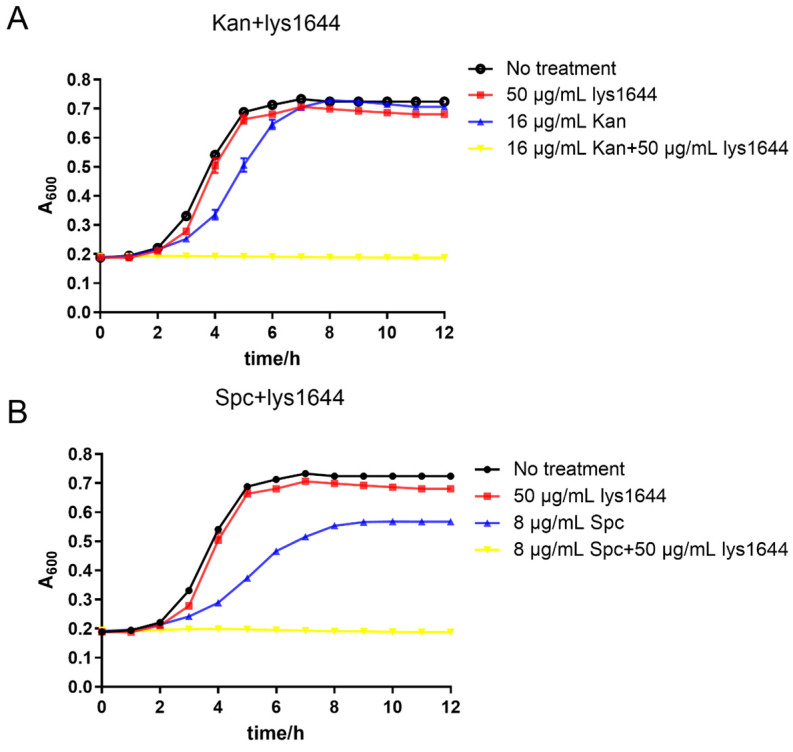
Synergistic effect of antibiotics and Lys1644. (**A**) Time-inhibit curves of *S. dysgalactiae* GS4-4 treated with Lys1644 (50 μg/mL), kanamycin (Kan, 1/4 MIC:16 μg/mL), or a combination of Lys1644 and Kan. (**B**) Time-inhibit curves of *S. dysgalactiae* GS4-4 treated with Lys1644 (50 μg/mL), spectinomycin (Spc, 1/4 MIC:8 μg/mL), or combinations of Lys1644 and Spc.

To explore the potential synergistic effect of Lys1644 in combination with antibiotics, we assessed the time-inhibit curves of the *S. dysgalactiae* GS4-4 strain treated with Lys1644 alone, antibiotics (kanamycin or spectinomycin), or a combination of Lys1644 and antibiotics. As shown in Figure 6A, the growth of bacteria could not be effectively inhibited to a stable stage by treatment with 50 μg/mL of Lys1644 or 16 μg/mL of kanamycin. However, the combination of Lys1644 and kanamycin completely inhibited bacterial growth. In addition, the combination of Lys1644 and spectinomycin also completely inhibited bacterial growth (Figure 6B). These results indicate that the combination of Lys1644 with kanamycin or spectinomycin exhibits a pronounced synergistic antibacterial effect.

## 3. Discussion

Antibiotic resistance has emerged as a significant challenge in the treatment of bovine mastitis, posing a potential threat to human health through the food chain [26]. Consequently, the development of a new generation of antimicrobials has become an urgent need. Phage lysins, characterized by a low probability of resistance development and a high effect against bacteria, offer a promising alternative to antibiotics [27,28]. Phage lysins are generally obtained by screening and sequencing the phages against specific bacteria. The virulent phages of *Streptococcus* are difficult to isolate, especially since no lytic phages against *S. dysgalactiae* have been reported so far [29,30,31]. However, some streptococcal genomes have integrated prophage genomes, which are either complete or incomplete. Some prophage genomes have genes encoding lysin, which is not harmful to bacteria during the lysogenic cycle due to strict regulation. It is an effective way to obtain the *streptococcus* lysin. The heterologous expression of lysin could verify its lytic activity against bacteria.

In this study, we obtained a lysin named Lys1644 from the prophage genome of a *S. dysgalactiae* clinical isolate. Notably, Lys1644 differs from previously reported streptococcal lysins such as PlyC, PlyPy, PlyGBS, B30, Pal, Clp-1 [13,14,15,16,17,18]. It possesses a specific and efficient lytic capability, showing stability within a certain pH and temperature range (Figure 3 and Figure 4; Table 1). Lys1644 can only kill *S. dysgalactiae* specifically, which ensures that it does not kill other bacteria, especially beneficial bacteria. This is where it has an advantage over antibiotics. Phage lysin could be applied for the prevention and control of mastitis in dairy cows through intra-milk infusion therapy and nipple dip disinfection in milking in dairy farms [12], so it is necessary to consider the activity of Lys1644 in milk. The activity of Lys1644 in milk is comparable to that in Tris-HCl (Figure 5), which indicates that it is a potential antibacterial agent in practical application.

A high dose of Lys1644 could result in a 2log10 CFUs/mL reduction in *S. dysgalactiae*, but was not suitable for practical use, so we explored the effect of a combination of low doses of Lys1644 with antibiotics. Antibiotics at concentrations below the minimum inhibitory concentration (MIC) or low doses of lysin alone were insufficient to completely inhibit the growth of bacteria. However, the combination of these agents demonstrated a significant synergistic effect (Figure 6). The antibiotics utilized in this study, kanamycin and spectinomycin, are both aminoglycoside antibiotics that can penetrate the bacterial cell and bind the 30S subunit of the ribosome to inhibit protein synthesis [32], whereas Lys1644 targets the peptidoglycan in the cell wall. A low dose of Lys1644 may not completely disrupt the peptidoglycan layer to cause bacterial death, but it can partially break the chemical bonds within peptidoglycan. This partial disruption leads to a more permeable structure, which in turn enhances the penetration of antibiotics. The use of a phage, its derived lysin, or depolymerase in conjunction with other antibacterial agents has demonstrated a synergistic effect, although the precise mechanism remains unclear [33,34]. While our hypothesis appears plausible, further investigation will be required in order to explore the specific synergy mechanism.

In this research, we introduce a newly discovered prophage lysin, Lys1644, that demonstrates potent bactericidal properties against *S. dysgalactiae.* Lys1644 has specific activity against *S. dysgalactiae*, high lytic activity in milk, and synergistic antibacterial effects with aminoglycoside antibiotics, which indicates that it might represent a potential therapeutic for the treatment of infections caused by *S. dysgalactiae*.

## 4. Materials and Methods

### 4.1. Bacteria and Plasmids

All bacterial strains and plasmids used in this study are listed in Table S1. *Streptococcus* strains were cultured in brain heart infusion medium (BHI, Qingdao Haibo, Qingdao, China) at 37 °C. *Escherichia coli* was cultured in Luria–Bertani broth medium (LB, Qingdao Haibo, Qingdao, China) at 37 °C.

### 4.2. Genome Sequencing and Prophage Analysis

The genomic DNA of *S. dysgalactiae* Lu24 was extracted and purified using the TIANamp Bacteria DNA Kit (Tiangen, Beijing, China). The quantity and quality of the DNA was measured using a NanoDrop Spectrophotometer 2000 (Equl-Thermo Scientific, Waltham, MA, USA). The DNA samples were sent to Personalbio (Shanghai, China) and subjected to mechanical fragmentation for the construction of a whole-genome shotgun library. The library was subjected to sequencing using the Illumina MiSeq platform to generate paired-end (2 × 250 bp) reads through next-generation sequencing (NGS) and the PacBio platform using third-generation single-molecule sequencing technology. Prophages in the genome were predicted by PhiSpy [35].

### 4.3. Structural Analysis of Lys1644

The gene *pp6_1644*, which was predicted to encode the lysin of the prophage pp6, was Lys1644. The amino acid sequence of Lys1644 was subjected to analysis using the Interpro server [36] and the AlphaFold2 server [37] to predict both the structure domain and the three-dimensional conformation.

### 4.4. Cloning of Lys1644

The gene *lys1644* was amplified by PCR using the following primers: AGGAGATATACCATGGGATCCATGACCTTTTTAGATAACAT (forward) and AAAATACAGGTTTTCGGTACCATTTAATTTACCCCAAAGAC (reverse). The gene *lys1644* was cloned into the expression plasmid pEC (carried a C-terminal 8×His tag) to construct the recombinant plasmid pEC-*lys1644* using a One-Step Cloning Kit (cat. number: C112-01, Vazyme, Nanjing, China). More information about pEC plasmids can be found in Figure S1 and Table S2. The pEC-*lys1644* vector was transformed into *E. coli* DH5α cells through transformation (Vazyme, Nanjing, China).

### 4.5. Recombinant Expression and Purification of Lys1644 Protein

The plasmid pEC-*lys1644* was transformed into *E. coli* BL21 (DE3) cells and cultured in LB medium supplemented with 50 μg/mL kanamycin at 37 °C. When the OD_600_ reached a range of 0.4 to 0.6, induction was initiated by adding 0.5 mM isopropyl-1-thio-β-D-galactopyranoside (IPTG), followed by cultivation at 25 °C for 16 h. Subsequently, the cells were collected, washed, subjected to ultrasonic disruption, and then purified using Ni-IDA affinity chromatography on a column (Sangon Biotech, Shanghai, China). Finally, the purified protein Lys1644 was analyzed via separation using sodium 12% dodecyl sulfate–polyacrylamide gel electrophoresis (SDS-PAGE) to check the purification efficiency and determine its molecular weight.

### 4.6. Lytic Activity of Lys1644

To test the lytic activity of Lys1644, *S. dysgalactiae* cells were adjusted to 0.6 of OD_600_ and subsequently treated with different final concentrations (0, 25, 50, 75, 100 μg/mL) of Lys1644 at 37 °C. The OD_600_ was measured at 10-minute intervals for 1 h. All reactions were performed in triplicate.

### 4.7. Antibacterial Spectrum of Lysin Lys1644

The different bacterial species (Table 1) were cultured until reaching the mid-log phase and adjusted to an OD_600_ of 0.6 using a 50 mM Tris-HCl solution. The bacteria were each mixed with lysin Lys1644 (final concentration: 50 μg/mL) and incubated at 37 °C for 1 h. Then, the mixture was subsequently subjected to spectrophotometer analysis using an ultraviolet spectrophotometer, TU-1810PC (PERSEE, Beijing, China), to measure the OD_600_. The decrease ratio of OD_600_ > 20% indicated the susceptibility of this strain to cleavage by Lys1644. All reactions were performed in triplicate.

### 4.8. Optimal pH of Lys1644

To test the optimal pH of Lys1644, *S. dysgalactiae* cells and Lys1644 were both prepared in 50 mM Tris-HCl buffer with varying pH values (ranging from 4 to 11). The cell cultures were adjusted to 0.6 of OD_600_. Subsequently, Lys1644 (final concentration: 50 μg/mL) was added to cells and incubated at 37 °C. After 1 h, the OD_600_ was measured. All reactions were performed in triplicate.

### 4.9. Optimal Temperature of Lys1644

To test the optimal temperature of Lys1644, *S. dysgalactiae* cells and Lys1644 were prepared in 50 mM Tris-HCl buffer at various temperatures (4, 25, 37, 42, and 56 °C). The cell cultures were adjusted to 0.6 of OD_600_. Subsequently, Lys1644 (final concentration: 50 μg/mL) was added to the cells and incubated at 37 °C for 1 h. After 1 h, the OD_600_ was measured. All reactions were performed in triplicate.

### 4.10. Lytic Activity of Lys1644 in Milk

To test the lytic activity of Lys1644 in milk, an equal number of *S. dysgalactiae* cells were prepared in milk and 50 mM Tris-HCl buffer (as control), respectively. Then, different final concentrations (0, 100, 200, 400, 800 μg/mL) of Lys1644 were added and incubated at 37 °C. After 1 h, serial dilutions of the mixture were performed, and subsequent plating on BHI solid medium was carried out. Following overnight incubation at 37 °C, the colonies were counted. All experiments were performed in triplicate.

### 4.11. Synergistic Bacteriostasis of Lys1644 and Antibiotics

The synergistic antibacterial effect of Lys1644 in combination with kanamycin and spectinomycin was selected for evaluation. Overnight-cultured *S. dysgalactiae* GS4-4 cells were inoculated into BHI medium at a 1/100 volume ratio and treated with Lys1644 (50 μg/mL), kanamycin (Kan, 1/4 of MIC:16 μg/mL), spectinomycin (Spc, 1/4 of MIC:8 μg/mL), combinations of Lys1644 and Kan, or combinations of Lys1644 and Spc. The A_600_ was measured every 1 h using a SpectraMax M2 microplate reader (Molecular Devices, San Jose, CA, USA) over a period of 12 h.

## Figures and Tables

**Table 1:** Activity spectrum of Lys1644.

Species	Strains	Lytic Effect
*S. dysgalactiae*	Lu24	+
	SD5-1	+
	GS4-4	+
*S. uberis*	HB19-2	−
	SX5-2	−
*S. agalactiae*	H11-1	−
*S. infantarius*	HB10-1	−
*S. suis*	TA16	−
*S. pneumoniae*	ATCC49619	−
*S. pneumoniae*	CCUG1407	−
*S. pyogenes*	ATCC12344	−
*L. monocytogenes*	EGDe	−
*S. aureus*	SA2	−
*E. faecalis*	V583	−
*E. faecium*	SX4-1	−
*E. coli*	ATCC25922	−

Note: “+” indicated can be lysed, “−” indicated can not be lysed.

## Data Availability

The authors confirm that all data underlying the findings are fully available without restriction. All relevant data are within the paper and its Supplementary Materials files (F‘igshare’ https://doi.org/10.6084/m9.figshare.25965196).

## Supplementary Materials

The following supporting information can be downloaded at: https://www.mdpi.com/article/10.3390/molecules29143411/s1, Figure S1: Map of pEC plasmid; Table S1: Bacteria strains and plasmids in this study; Table S2: Base sequence of pEC; Table S3: Sequence of Lys1644.
