# Supplementary material for: Identification and Characterization of a Novel Prophage Lysin against Streptococcus dysgalactiae
Source: Molecules. 2024 Jul 20;29(14):3411. doi: 10.3390/molecules29143411 (PMC11279900; doi:10.3390/molecules29143411)
Supplement: Supplementary file 1 [file molecules-29-03411-s001.zip › molecules-3067125-supplementary.pdf]

Figure S1. Map of pEC plasmid

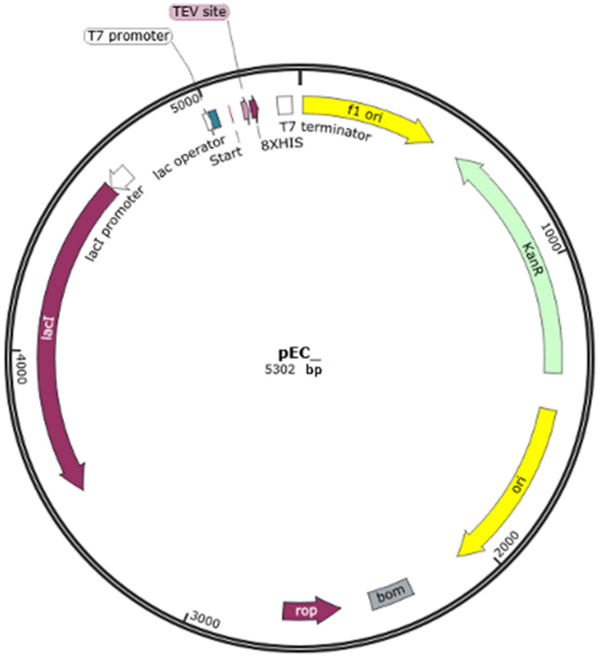

Table S1. Bacteria strains and plasmids in this study.

Strains	Description	Source
E. coli DH5α	Host for cloning	Purchased from Vazyme
E. coli BL21(DE3)	Host for protein expression	Purchased from Vazyme
Lu24	*S. dysgalactiae* wild type	A dairy farm from Dezhou, Shandong, China
SD5-1	*S. dysgalactiae* wild type	A dairy farm from Dezhou, Shandong, China
GS4-4	*S. dysgalactiae* wild type	A dairy farm from Wuwei, Gansu, China
HB19-2	*S. uberis* wild type	A dairy farm from Xingtai, Hebei, China
SX5-2	*S. uberis* wild type	A dairy farm from Datong, Shanxi, China
H11-1	*S. agalactiae* wild type	A dairy farm from Zhangjiakou, Hebei, China
HB10-1	*S. infantarius* wild type	A dairy farm from Xingtai, Hebei, China
TA16	*S. suis* wild type	A pig farm from Taian, Shandong, China
ATCC49619	*S. pneumoniae* wild type	Our lab
CCUG1407	*S. pneumoniae* wild type	Our lab
ATCC12344	*S. pyogenes* wild type	Our lab
EGDe	*L. monocytogenes* wild type	Our lab
SA2	*S. aureus* wild type	Our lab

V583	*E. faecalis* wild type	Our lab
SX4-1	*E. faecium* wild type	Our lab
ATCC25922	*E. coli* wild type	Our lab
Plasmids		
pEC	Vector for protein expression; Kanamycin resistance; Carrying 6×His tag	A gift from the National Center for Protein Science (Shanghai).

Table S2. Base sequence of pEC

Base sequence of pEC
TGGCGAATGGGACGCGCCCTGTAGCGGCGCATTAAGCGCGGCGGGTGTG<br>GTGGTTACGCGCAGCGTGACCGCTACACTTGCCAGCGCCCTAGCGCCCG<br>CTCCTTTCGCTTTCTTCCCTTCCTTTCTCGCCACGTTCGCCGGCTTTCCCC<br>GTCAAGCTCTAAATCGGGGGCTCCCTTTAGGGTTCCGATTTAGTGCTTTA<br>CGGCACCTCGACCCCAAAAAACTTGATTAGGGTGATGGTTCACGTAGTG<br>GGCCATCGCCCTGATAGACGGTTTTTCGCCCTTTGACGTTGGAGTCCACG<br>TTCTTTAATAGTGGACTCTTGTTCCAAACTGGAACAACACTCAACCCTAT<br>CTCGGTCTATTCTTTTGATTTATAAGGGATTTTGCCGATTTCGGCCTATTGG<br>TTAAAAAATGAGCTGATTTAACAAAAATTTAACGCGAATTTTAACAAAAT<br>ATTAACGTTTACAATTTCAGGTGGCACTTTTCGGGGAAATGTGCGCGGAA<br>CCCCTATTTGTTTATTTTTCTAAATACATTCAAATATGTATCCGCTCATGAAT<br>TAATTCTTAGAAAAACTCATCGAGCATCAAATGAAACTGCAATTTATTCAT<br>ATCAGGATTATCAATACCATATTTTTGAAAAAGCCGTTTCTGTAATGAAGG<br>AGAAAACTCACCGAGGCAGTTCCATAGGATGGCAAGATCCTGGTATCGG<br>TCTGCGATTCCGACTCGTCCAACATCAATACAACCTATTAATTTCCCCTCG<br>TCAAAAATAAGGTTATCAAGTGAGAAATCACCATGAGTGACGACTGAAT<br>CCGGTGAGAATGGCAAAAGTTTATGCATTTCTTTCCAGACTTGTTCAACA<br>GGCCAGCCATTACGCTCGTCATCAAAATCACTCGCATCAACCAAACCGTT<br>ATTCATTCGTGATTGCGCCTGAGCGAGACGAAATACGCGATCGCTGTTAA<br>AAGGACAATTACAAACAGGAATCGAATGCAACCGGCGCAGGAACACTG<br>CCAGCGCATCAACAATATTTTCACCTGAATCAGGATATTCTTCTAATACCT<br>GGAATGCTGTTTTCCCGGGGATCGCAGTGGTGAGTAACCATGCATCATCA<br>GGAGTACGGATAAAATGCTTGATGGTCGGAAGAGGCATAAATTCCGTCA<br>GCCAGTTTAGTCTGACCATCTCATCTGTAACATCATTGGCAACGCTACCTT<br>TGCCATGTTTCAGAAACAACTCTGGCGCATCGGGCTTCCCATACAATCGA<br>TAGATTGTCGCACCTGATTGCCCGACATTATCGCGAGCCCATTTATACCCA<br>TATAAATCAGCATCCATGTTGGAATTTAATCGCGGCCTAGAGCAAGACGT<br>TTCCCGTTGAATATGGCTCATAACACCCCTTGTATTACTGTTTATGTAAGC<br>AGACAGTTTTATTGTTCATGACCAAAATCCCTTAACGTGAGTTTTCGTTCC<br>ACTGAGCGTCAGACCCCGTAGAAAAGATCAAAGGATCTTCTTGAGATCC<br>TTTTTTTCTGCGCGTAATCTGCTGCTTGCAAACAAAAAAACCACCGCTAC<br>CAGCGGTGGTTTGTTTGCCGGATCAAGAGCTACCAACTCTTTTTCCGAAG<br>GTAACTGGCTTCAGCAGAGCGCAGATACCAAATACTGTCCTTCTAGTGTA<br>GCCGTAGTTAGGCCACCACTTCAAGAACTCTGTAGCACCGCCTACATACC

TCGCTCTGCTAATCCTGTTACCAGTGGCTGCTGCCAGTGGCGATAAGTCG
TGTCTTACCGGGTTGGACTCAAGACGATAGTTACCGGATAAGGCGCAGC
GGTCGGGCTGAACGGGGGGTTCGTGCACACAGCCCAGCTTGGAGCGAA
CGACCTACACCGAACTGAGATACCTACAGCGTGAGCTATGAGAAAGCGC
CACGCTTCCCGAAGGGAGAAAGGCGGACAGGTATCCGGTAAGCGGCAG
GGTCGGAACAGGAGAGCGCACGAGGGAGCTTCCAGGGGGAAACGCCTG
GTATCTTTATAGTCCTGTCGGGTTTCGCCACCTCTGACTTGAGCGTCGATT
TTTGTGATGCTCGTCAGGGGGGCGGAGCCTATGGAAAAACGCCAGCAAC
GCGGCCTTTTTACGGTTCCTGGCCTTTTGCTGGCCTTTTGCTCACATGTTC
TTTCCTGCGTTATCCCCTGATTCTGTGGATAACCGTATTACCGCCTTTGAG
TGAGCTGATACCGCTCGCCGCAGCCGAACGACCGAGCGCAGCGAGTCA
GTGAGCGAGGAAGCGGAAGAGCGCCTGATGCGGTATTTTCTCCTTACGC
ATCTGTGCGGTATTTCACACCGCATATATGGTGCACTCTCAGTACAATCTG
CTCTGATGCCGCATAGTTAAGCCAGTATACACTCCGCTATCGCTACGTGAC
TGGGTCATGGCTGCGCCCCGACACCCGCCAACACCCGCTGACGCGCCCT
GACGGGCTTGTCTGCTCCCGGCATCCGCTTACAGACAAGCTGTGACCGT
CTCCGGGAGCTGCATGTGTCAGAGGTTTTCACCGTCATCACCGAAACGC
GCGAGGCAGCTGCGGTAAAGCTCATCAGCGTGGTCGTGAAGCGATTCAC
AGATGTCTGCCTGTTCATCCGCGTCCAGCTCGTTGAGTTTCTCCAGAAGC
GTTAATGTCTGGCTTCTGATAAAGCGGGCCATGTTAAGGGCGGTTTTTTC
CTGTTTGGTCACTGATGCCTCCGTGTAAGGGGGATTTCTGTTCATGGGGG
TAATGATACCGATGAAACGAGAGAGGATGCTCACGATACGGGTTACTGAT
GATGAACATGCCCGGTTACTGGAACGTTGTGAGGGTAAACAACTGGCGG
TATGGATGCGGCGGGACCAGAGAAAAATCACTCAGGGTCAATGCCAGCG
CTTCGTTAATACAGATGTAGGTGTTCCACAGGGTAGCCAGCAGCATCCTG
CGATGCAGATCCGGAACATAATGGTGCAGGGCGCTGACTTCCGCGTTTCC
AGACTTTACGAAACACGGAAACCGAAGACCATTCATGTTGTTGCTCAGG
TCGCAGACGTTTTGCAGCAGCAGTCGCTTCACGTTCGCTCGCGTATCGGT
GATTCATTCTGCTAACCAGTAAGGCAACCCCGCCAGCCTAGCCGGGTCCT
CAACGACAGGAGCACGATCATGCGCACCCGTGGGGCCGCCATGCCGGCG
ATAATGGCCTGCTTCTCGCCGAAACGTTTGGTGGCGGGACCAGTGACGA
AGGCTTGAGCGAGGGCGTGCAAGATTCCGAATACCGCAAGCGACAGGC
CGATCATCGTCGCGCTCCAGCGAAAGCGGTCCTCGCCGAAAATGACCCA
GAGCGCTGCCGGCACCTGTCCTACGAGTTGCATGATAAAGAAGACAGTC
ATAAGTGCGGCGACGATAGTCATGCCCCGCGCCCACCGGAAGGAGCTGA
CTGGGTTGAAGGCTCTCAAGGGCATCGGTCGAGATCCCGGTGCCTAATG
AGTGAGCTAACTTACATTAATTGCGTTGCGCTCACTGCCCGCTTTCCAGT
CGGGAAACCTGTCGTGCCAGCTGCATTAATGAATCGGCCAACGCGCGGG
GAGAGGCGGTTTGCGTATTGGGCGCCAGGGTGGTTTTTCTTTTCACCAGT
GAGACGGGCAACAGCTGATTGCCCTTCACCGCCTGGCCCTGAGAGAGTT
GCAGCAAGCGGTCCACGCTGGTTTGCCCCAGCAGGCGAAAATCCTGTTT
GATGGTGGTTAACGGCGGGATATAACATGAGCTGTCTTCGGTATCGTCGT
ATCCCACTACCGAGATATCCGCACCAACGCGCAGCCCGGACTCGGTAATG
GCGCGCATTGCGCCCAGCGCCATCTGATCGTTGGCAACCAGCATCGCAG

TGGGAACGATGCCCTCATTCAGCATTTGCATGGTTTGTTGAAAACCGGAC<br>ATGGCACTCCAGTCGCCTTCCCGTTCCGCTATCGGCTGAATTTGATTGCG<br>AGTGAGATATTTATGCCAGCCAGCCAGACGCAGACGCGCCGAGACAGAA<br>CTTAATGGGCCCGCTAACAGCGCGATTTGCTGGTGACCCAATGCGACCA<br>GATGCTCCACGCCCAGTCGCGTACCGTCTTCATGGGAGAAAATAATACTG<br>TTGATGGGTGTCTGGTCAGAGACATCAAGAAATAACGCCGGAACATTAG<br>TGCAGGCAGCTTCCACAGCAATGGCATCCTGGTCATCCAGCGGATAGTTA<br>ATGATCAGCCCACTGACGCGTTGCGCGAGAAGATTGTGCACCGCCGCTT<br>TACAGGCTTCGACGCCGCTTCGTTCTACCATCGACACCACCACGCTGGCA<br>CCCAGTTGATCGGCGCGAGATTTAATCGCCGCGACAATTTGCGACGGCGC<br>GTGCAGGGCCAGACTGGAGGTGGCAACGCCAATCAGCAACGACTGTTT<br>GCCCGCCAGTTGTTGTGCCACGCGGTTGGGAATGTAATTCAGCTCCGCCA<br>TCGCCGCTTCCACTTTTTCCCGCGTTTTCGCAGAAACGTGGCTGGCCTGG<br>TTCACCACGCGGGAAACGGTCTGATAAGAGACACCGGCATACTCTGCGA<br>CATCGTATAACGTTACTGGTTTCACATTCACCACCCTGAATTGACTCTCTT<br>CCGGGCGCTATCATGCCATACCGCGAAAGGTTTTGCGCCATTCGATGGTG<br>TCCGGGATCTCGACGCTCTCCCTTATGCGACTCCTGCATTAGGAAGCAGC<br>CCAGTAGTAGGTTGAGGCCGTTGAGCACCGCCGCCGCAAGGAATGGTGC<br>ATGCAAGGAGATGGCGCCCAACAGTCCCCCGGCCACGGGGCCTGCCACC<br>ATACCCACGCCGAAACAAGCGCTCATGAGCCCGAAGTGGCGAGCCCGAT<br>CTTCCCCATCGGTGATGTCGGCGATATAGGCGCCAGCAACCGCACCTGTG<br>GCGCCGGTGATGCCGGCCACGATGCGTCCGGCGTAGAGGATCGAGATCT<br>CGATCCCGCGAAATTAATACGACTCACTATAGGGGAATTGTGAGCGGATA<br>ACAATTCCCCTCTAGAAATAATTTTGTTTAACTTTAAGAAGGAGATATACC<br>ATGGGATCCAAGCTTAGCGGCCGCGAATTCCTCGAGGGTACCGAAAACC<br>TGTATTTTCAGGGCTCTGGACAtCAtCACCACCACCACCACCACTGAGATC<br>CGGCTGCTAACAAAGCCCGAAAGGAAGCTGAGTTGGCTGCTGCCACCG<br>CTGAGCAATAACTAGCATAACCCCTTGGGGCCTCTAAACGGGTCTTGAGG<br>GGTTTTTTGCTGAAAGGAGGAACTATATCCGGAT

Table S3. Sequence of Lys1644

Amino acid sequence of Lys1644
MTFLDNIKQGCLDGWAKYKILPSLTAAQAILESGWGKHAPHNALFGIKADS<br>SWAGKSFNTKTQEEYQPGVMTDIVDRFRAYDSWTDSIFDHGKFLNDNPRY<br>QAVVGETDYKKACHAIKDAGYATASGYAELLIQLIEENDLQKWDDEAIGGK<br>EKQMISSQCREVIEFFINLANAGMGVDKDNFAGWQCADVPCYAAKHWFG<br>VDLWGNAIDLLDSAAAAGWEVHRMPTDANPRAGAFFVQSVPYHQFGHTG<br>IVIEDSDGYTMRTIEQNIDGNADALYVGAPARFNTRDFTGVIGWFYPPYQGD<br>AVTQTVSTEPQTSDTIVETPKSGTFTLDVAEINIRRWPSLASEVVGSYKQGDT<br>VGFDSEGYANGYYWISYVGGSGKRNYLAIGQTDKDGNRISLWGKLN
